# Decreased USP2a Expression Inhibits Trophoblast Invasion and Associates With Recurrent Miscarriage

**DOI:** 10.3389/fimmu.2021.717370

**Published:** 2021-08-19

**Authors:** Jiayu Wang, Jinli Ding, Sainan Zhang, Xin Chen, Sisi Yan, Yan Zhang, Tailang Yin

**Affiliations:** ^1^Reproductive Medicine Center, Renmin Hospital of Wuhan University, Wuhan, China; ^2^Department of Clinical Laboratory, Renmin Hospital of Wuhan University, Wuhan, China

**Keywords:** USP2a, trophoblast invasion, recurrent miscarriage, decidual macrophage, TGF-β

## Abstract

An appropriate development of the placenta consisting of trophoblast cell migration, invasion, proliferation, and apoptosis, is essential to establishing and maintaining a successful pregnancy. Ubiquitin‐specific protease 2a (USP2a) regulates the processes of metastasis in multiple tumor cells. Yet, no known research has focused on exploring the effect of USP2a on trophoblasts and its possible mechanism in the pathogenies of recurrent miscarriage (RM). In this study, we first detected the decreased mRNA levels and the protein levels of USP2a in placental villous tissue samples from the RM patients. *In vitro* assays verified that overexpression of USP2a promoted human trophoblast proliferation, migration, invasion, whereas knockdown of USP2a inhibited these processes. Mechanistically, USP2a activated PI3K/Akt/GSK3β signaling pathway to promote nuclear translocation of β‐catenin and further activated epithelial-mesenchymal transition (EMT) in the trophoblasts. Moreover, transforming growth factor-beta (TGF-β) up-regulated USP2a expression in trophoblasts. Interestingly, M2 macrophage secreted TGF-β induced trophoblast migration and invasion, and an anti-TGF-β antibody alleviated this effect. Collectively, this study indicated that USP2a regulated trophoblast invasion and that abnormal USP2a expression might lead to aberrant trophoblast invasion, thus contributing to RM.

## Introduction

RM is defined by the consecutive loss of two or more clinical pregnancies (1) and affects approximately 2-5% of couples at reproductive age (2). RM as a severe global health issue, carries a heavy psychological and financial burden for affected couples. As one of the prerequisites for a successful pregnancy, adequate placentation relies on a delicate chorus between fetal trophoblasts and maternal cells, including proper maternal-fetal crosstalk and trophoblasts development (3). Deep invasion of placental trophoblast cells into the maternal decidua and myometrium is essential for placental embedment and fetal development. Epithelial-mesenchymal transition (EMT), characterized by loss of adhesive epithelial phenotype and acquisition of motile mesenchymal phenotype, has been proven to significantly promote the migration and invasion of extravillous trophoblast cells (EVTs) (4–6). Reduced trophoblast proliferation, excessive trophoblast cell apoptosis, along with insufficient trophoblast invasion have all been tightly linked to the development of RM (7). Thus, exploring trophoblast development is crucial to further understanding RM pathogenesis.

Trophoblast invasion is an intricate process involving interactions with stromal cells, glands, arteries, and immune cells, including decidual natural killer cells, macrophages, dendritic cells, T cells, and a range of cytokines and chemokines (8). During the establishment and maintenance of pregnancy, the well-orchestrated crosstalk between fetal trophoblasts and maternal immune cells facilitates the formation of a functional placenta (9). Thereby, successful pregnancy requires a robust and highly dynamic immune system. Post-implantation decidua is rich in immune infiltrates (10). Macrophages are the second-largest immune cell group and comprise 20–25% of all immune cells at the maternal-fetal interface (11, 12). Accumulating evidence indicates that decidual macrophages are skewed toward an M2-like phenotype involving tissue remodeling, cell proliferation, and generation of immunosuppressive microenvironments in the early pregnancy uterus (12). Previous studies have shown that macrophages can establish “crosstalk” with trophoblasts in the maternal-fetal interface microenvironment *via* a complex cytokine-based connection. In addition, macrophages can secrete amounts of soluble mediators to regulate the biological behaviors of trophoblasts, including IL-10, TGF-β, IL-4, and VEGF (13–15). Also, macrophages can respond to various factors produced by trophoblasts to regulate polarization status, thereby exerting different biological functions. Previously, our group has demonstrated that IL-6 secreted by trophoblasts could induce macrophages polarization to M2 phenotype, thereby modulating the process of early pregnancy (16). However, understanding the abnormal interactions between trophoblasts and decidua macrophages at the maternal-fetal interface and its contribution to RM remains limited.

Ubiquitination, as one of the important post-translational modifications, can selectively attach ubiquitin to its substrate to regulate protein degradation, activity, and protein-protein interaction as well (17). The ubiquitin system is highly dynamic and tightly controlled by the family of deubiquitinating enzymes specifically involved in removing ubiquitin molecules on target proteins (18). The deubiquitinating enzyme USP2a inhibits the ubiquitination of FAS, MDM2, cyclin, MYC, Aurora A and regulates their stability and function, thereby participating in the regulation of the metabolism, development, apoptosis, cell proliferation, and differentiation of tumor cells (19–22). Recent studies have shown that USP2a removes the K33-linked ubiquitin chain at Lys502 of TGFBR1 to promote the recruitment and phosphorylation of SMAD2/3, promoting the transcription of EMT-related genes induced by TGF-β (23). In addition, USP2a promotes the nuclear accumulation of β-catenin and enhances its transcriptional activity by deubiquitinating β-catenin, and activates the Wnt/β-catenin signaling pathway (24). Given the similar regulatory mechanism and biological characteristics of EVTs and tumor cells, we propose whether USP2a participates in the occurrence of RM by regulating the invasive ability of EVTs at the maternal-fetal interface.

Here in the current study, we firstly reported that USP2a facilitated the proliferation, invasion, and migration of trophoblasts by activating the PI3K/Akt/GSK3β/β-catenin signaling pathway. Furthermore, USP2a expression is regulated positively by TGF-β derived from M2 macrophages. In addition, USP2a expression was down-regulated in chorionic villi of RM patients. Thus, our results suggest that USP2a is a trophoblast proliferation and invasion-associated enzyme that might participate in RM’s pathogenesis.

## Materials and Methods

### Patients and Tissue Samples

In this study, women with normal pregnancies who chose to undergo an artificial abortion due to their voluntary willingness were selected as the healthy control group (NC). Patients with unexplained recurrent spontaneous abortions two or more times were regarded as the RM group (RM). This study enrolled a total of 24 RM patients treated in Renmin Hospital of Wuhan University and 28 normal pregnant women. All patients denied having a history of chronic diseases such as hypertension and diabetes. RM patients with the following characteristics were excluded: abnormal anatomical structure of the reproductive tract; infection of the reproductive tract; reproductive endocrine disease; prethrombotic state; fetal chromosomal abnormality. The patients in the control group had a history of more than one live birth, and there was no history of adverse pregnancy, such as spontaneous abortion, eclampsia, and preterm birth. These patients received an abortion between 7 and 9 weeks of gestation to terminate the unintended pregnancy. During the operation, samples of placental chorionic and decidual tissues were collected or fixed with 4% paraformaldehyde and embedded in paraffin in blocks or frozen and stored in liquid nitrogen. The procedure was carried out under the approval of the internal review and ethics committee of the Renmin Hospital of Wuhan University, and the informed consent of all patients was obtained.

### Cell Culture and Reagents

The HTR-8/SVneo cell line and BeWo cell line were obtained from China Center for Type Culture Collection. HTR-8 and BeWo were respectively cultivated in Dulbecco’s modified Eagle’s minimal essential medium (DMEM)/F12 (Gibco, USA) and Minimum Essential Medium Eagle medium (MEM) (Gibco, USA) with 10% fetal bovine serum (FBS) (Gibco, USA) and 1% antibiotic, at 37°C in the presence of 5% CO_2_.

For macrophage generation, the human monocyte cell line THP-1 was cultured and grown in RPMI-1640 medium (Gibco, USA) with 10% FBS. THP-1 cells were treated with 100 ng/ml Phorbol1 2-myristate 13-acetate (PMA; Sigma, USA) for 24 h. Macrophages were polarized in M1 macrophages by incubation with 20 ng/ml of IFN-γ (Peprotech, USA) and 100 ng/ml of LPS (Peprotech, USA). Macrophage M2 polarization was obtained by incubation with 20 ng/ml of IL-4 (Peprotech, USA) and 20 ng/ml of IL-13 (Peprotech, USA). Macrophages and trophoblasts co-cultivation was conducted using the non-contact co-culture transwell system (Corning, USA) for 72h, in which macrophages were seeded in 0.4 μm-sized pores inserts and the trophoblast cells were seeded in the 6-well plate.

Recombinant human TGF-β1 (Peprotech, USA) was dissolved in ddH2O and used at a final concentration of 25ng/mL. LY294002 (PI3K inhibitors) was purchased from Med Chem Express, China. The anti-human neutralizing TGF-β antibody was acquired from R&D Systems, USA.

### Overexpression of USP2a

To generate the USP2a-overexpressing construct, we cloned the coding region sequence of human USP2a into the pLVX-IRES-ZsGreen vector (Vigene) using the following primers: 5’-CGCAAATGGGCGGTAGGCGTG-3’ (forward) and 5’-CCTCTACAAATGTGGTATGGC-3’ (reverse). All constructs were verified by sequencing and transfected into the cells using Lipofectamine 3000 (Invitrogen). Forty-eight hours after transfection, cells were collected for RT-PCR, western blot, migration, and invasion assays.

### Knockdown of USP2a

shRNA was designed explicitly targeting the USP2a, and the sequence was as follows: 5’-GCATGAGGCTCTTTTCACCAA-3’. Empty vector or lentivirus packaging shRNA was transfected in HEK293T cells. After 8 hours of transfection, the cell culture medium was changed to a complete medium. After 48 hours, the virus-containing supernatant was collected and filtered with a 0.22um filter to infect the trophoblast cell line mentioned above. After 24h, Puromycin was added for screening to construct a stably transfected cell line.

### Cell Proliferation Assay

Cell proliferation was assessed using a Cell Counting Kit-8 (CCK-8, Beyotime, Shanghai, China) and EdU assay (Abcam, USA). Trophoblast cells were plated at 2×10^3^ cells/well in 96-well plates. Cell proliferation ability was determined at 24, 48, 72, or 96 hours using the CCK-8. At the above time point, the supernatant was removed, 10% CCK-8 solution was added to each well, and the cells were cultured for two more hours. The enzyme labeling instrument measured the absorbance at 450 nm wavelength. EdU experiments were carried out to analyze the proliferation of HTR-8 and BeWo cells according to the manufacturer’s instructions. The percentage of EdU positive cells (EdU positive/DAPI positive) was labeled as EdU positive cells. Five technical replicates were done per experiment, and three independent experiments were performed. Colony formation assay was performed as described. After transfection, cells were seeded to six-well plates at densities ranging from 100 to 40,000 cells per well and were put in an incubator for 10–14 days. After incubation at 37°C, cells were fixed with glutaraldehyde and stained with crystal violet. Colony formation for each condition was compared with that of untreated control cells. Results were obtained from five replicate wells, and each experiment was repeated three times.

### Wound-Healing Assay

The wound-healing assay was used to evaluate the migration ability of trophoblasts. Cells in the fresh medium were placed into a 6-well plate chamber (5×10^5^ cells/well) and incubated for 24 h. When the cells reached 80%-90% confluence, a scratch across the cell surface was made by the plastic pipette tip scraped across the cell surface. Phosphate-buffered saline (PBS) was used to remove floating debris, and the wound was photographed immediately (0 h). The cells were then cultured in the serum-free medium. The wounds were photographed at 24 h and 48 h to measure the extent of wound healing. The sizes of the gaps were measured with Image-Pro Plus software.

### Transwell Invasion Assay

Trypsin Solution (Biosharp, China) was used to collect the transfected trophoblast cells. 5×10^4^ cells in 100 µl FBS-free medium were seeded into the upper chamber of the transwell insert with 8 µm pores (Corning Costar Corp, Corning, NY, USA) which was precoated with matrix Matrigel (BD Biosciences, CA, USA) to evaluate the invasion ability of the trophoblast cells. The transwell chambers without matrix Matrigel were performed to assess the migration ability. The lower chamber was filled by medium supplemented with 10% FBS (600 µl), and the apparatus was incubated at 37°C. After two days, the cells in the insert were fixed using 4% paraformaldehyde for thirty minutes and stained using 0.5% crystal violet. Five random fields under the microscope were used to calculate the number of invaded cells.

### Apoptosis Detection Using Annexin V-PE/7-AAD Double Staining

Plasma membrane permeability and phosphatidylserine cell translocation were measured by flow cytometry to define the percentage of apoptotic cells. To this aim, dual staining was performed with PE or APC-conjugated Annexin-V and 7-amino-actinomycin D (7AAD) using the Annexin-V/7AAD apoptosis detection kit (BD Biosciences). The adherent cells were collected. After washed two times with cold PBS, the cells were resuspended in 100 µl PBS. The Cell suspension was co-stained with 10 µl 7-AAD and 5 µl Annexin V-PE in the dark for 15 minutes at room temperature before analysis using flow cytometry. Early apoptotic cells were defined as Annexin-V+/7AAD− cells and Annexin-V+/7AAD+ as late apoptotic cells. Results were analyzed by flow cytometry (FACS Calibur, BD).

### RNA Isolation and RT–PCR

Total RNA was extracted using the TRIzol RNA purification kit, and NanoDrop 2000 spectrophotometer was used to evaluate RNA purity and quantification. One μg of the total RNA was used to synthesize cDNA using the Takara PrimeScript RT reagent kit according to the manufacturer’s instructions. Gene expression was determined by RT–PCR using SYBR Green Supermix (Takara, Dalian, China). A cycle threshold (CT) value was obtained for each sample, and duplicate sample values were averaged. The 2^-ΔΔCT^ method was used to calculate relative expression of each target gene (25) Briefly, mean CT value of target genes in each sample was normalized to its averaged housekeeping gene CT value to give a ΔCT value. This was then normalized to control samples (ΔΔCT), and the 2^-ΔΔCT^ value was obtained. The mRNA expression level under a given condition was quantified following normalization to the internal control GAPDH (mean±SEM).

### Immunohistochemical and Immunofluorescence Staining

Immunohistochemical staining of human villi or placenta tissue was performed as previously described using rabbit anti-USP2 (dilution 1:200; Abgent, UK), rabbit anti-Cytokeratin 7 (CK7) (dilution 1:200; proteintech), rabbit anti-HLA-G (dilution 1:200; proteintech). Cells were fixed in 4% paraformaldehyde, permeabilized using 1% Triton X-100 for 30 min, and blocked with 5% BSA for one hour at room temperature. Indirect immunofluorescence was performed using primary antibodies, rabbit anti–β-catenin (1:1,000; Cell Signaling), and secondary antibodies, goat anti-rabbit (1:1,000; proteintech). Nuclear were counterstained with 4′,6-diamindino-2-phenylindole (DAPI; Molecular Probes). Images were acquired using an Olympus microscope. The confocal images were acquired by Laser Scanning Biological Microscope (Olympus). Immunohistochemical staining quantifications were assessed using ImageJ software.

### Western Blotting and Co-IP Assay

Western blotting was used to determine protein levels. Protein concentrations were determined using a BCA Protein Assay Kit (Beyotime), according to the manufactures’ instructions. SDS-PAGE was used to separate the proteins, which were then electrotransferred onto polyvinylidene difluoride (PVDF) membranes (Millipore, USA). After blocking the membranes using 5% non-fat dry milk in TBST for 1.5 h, the blots were incubated at 4°C overnight with various primary rabbit antibodies. The membranes were washed with TBST four times (5 minutes each time) and then incubated with secondary antibodies at room temperature for one hour. The membranes were then washed with TBST four times. Finally, the blots were developed using ChemiDoc™ Imager from Bio‐Rad. The level of GAPDH was used as a loading control to normalize the specific protein levels.

For co-IP, cell extracts were prepared by brief sonication in IP buffer (50 mM Tris–HCl, pH 8.0, 150 mM NaCl, 2 mM EDTA, 0.5% NP‐40, 5% glycerol). The samples were incubated with1–2 μg of antibodies overnight, and 30 μl of a 25% protein A+G agarose (ThermoFisher, USA) was added. Three-four hours later, beads were washed with IP buffer three times, and bound proteins were detected by Western blot.

The following primary antibodies were used: anti-USP2 (C-term L523) (Abgent, UK), anti‐E‐cadherin (Proteintech, Chicago), vimentin (Proteintech, Chicago), Snail (Proteintech, Chicago), and anti‐N‐cadherin (Proteintech, Chicago). In addition, rabbit polyclonal antibodies, including those recognizing PI3K, phosphorylated (p)- PI3K, AKT, p-AKT, GSK3β, β-catenin, BAX, BCL2, and glyceraldehyde 3-phosphate dehydrogenase (GAPDH), were obtained from Cell Signaling Technology (Danvers, MA, USA). The antibodies were used at a working concentration of 1:1000 and were stored at 4°C. The secondary antibodies were purchased from LI-COR (Lincoln, NE, USA) and used at a dilution ratio of 1:10,000.

### Statistical Analysis

Statistical analyses were performed using SPSS 20 (IBM Corp, USA) and GraphPad Prism 8.0 (GraphPad Software Inc, USA) for Windows. Statistical significance was accepted at a value of *P* < 0.05. All experiments were repeated three times. *In vitro* experiments were analyzed using unpaired Student’s *t*-test and ordinary one-way ANOVA test for multiple comparisons.

## Results

### The Expression and Localization of USP2a in Placental Villous Tissues

**Figure 1:**
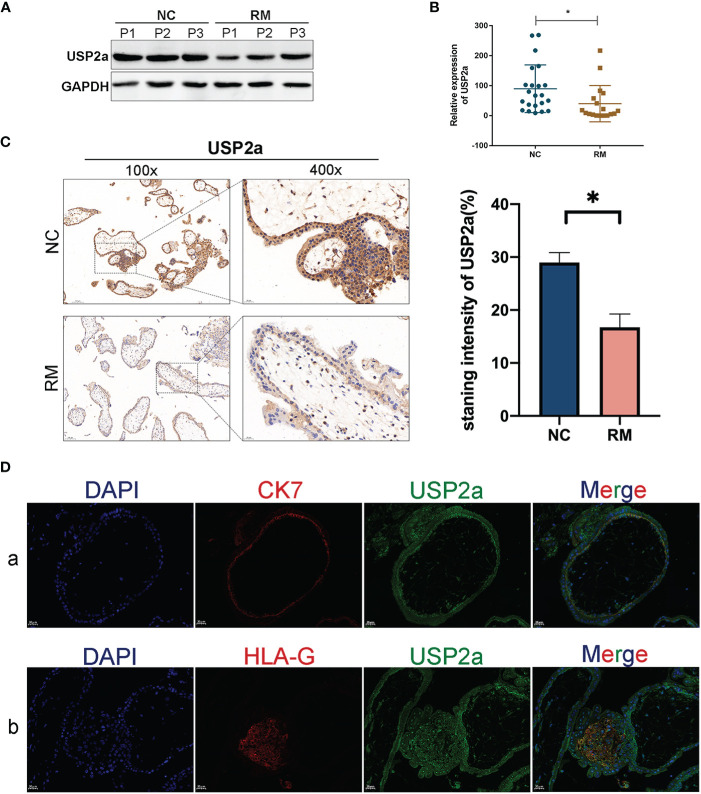
USP2a is down-regulated in placental in patients with recurrent miscarriage (RM). **(A, B)** Comparison of relative mRNA and protein expression levels of USP2a in placental villous tissues between RM patients (n = 18) and normal women (n = 22) by RT‐PCR and western blot. **(C)** Representative immunohistochemical images of villous expression and localization of USP2a in RM patients (n = 22) and normal women (n = 25). The staining intensity was quantified using the ImageJ software. Graphs show the means ± SD. Magnification, 100× and 400×. **(D)** Representative immunofluorescence images: (a) double immune-staining of CK7 (red) and USP2a (green). Magnification, 400×; (b) double immune-staining of HLA-G (red) and USP2a (green) Magnification, 400×. *P < 0.05. Scale bar = 100 μm (100×); Scale bar = 20 μm (400×). mRNA, messenger RNA; RT‐PCR, quantitative real‐time polymerase chain reaction; RM, recurrent miscarriage; USP2a, ubiquitin‐specific protease 2a; CK7, cytokeratin 7; HLA-G, human leukocyte antigen G.

RT-PCR and Western blot analysis of first-trimester chorionic villous tissue was performed to explore whether USP2a is involved in the pathogenesis of RM. USP2a expression was down-regulated significantly in the villous tissue of patients with RM (**Figures 1A, B** **)**. Furthermore, immunohistochemical analysis of paraffin-embedded tissue was performed to investigate USP2a localization in the first-trimester villous further. Stronger expression of USP2a was observed in tissue of the NC group, with the positive cells mainly being cytotrophoblasts and EVTs (**Figure 1C**). To verify the above result, we respectively used immunofluorescence staining with antibodies of CK7 as a marker of cytotrophoblasts, and HLA-G, as a marker of EVTs on human villous tissue, as shown in **Figure 1D**. Immunofluorescence shows resemblant results. The above results demonstrated the aberrant expression of USP2a might be involved in the pathogenesis of RM.

### The Influences of USP2a on the Proliferation and Apoptosis Ability of Trophoblast Cells

**Figure 2:**
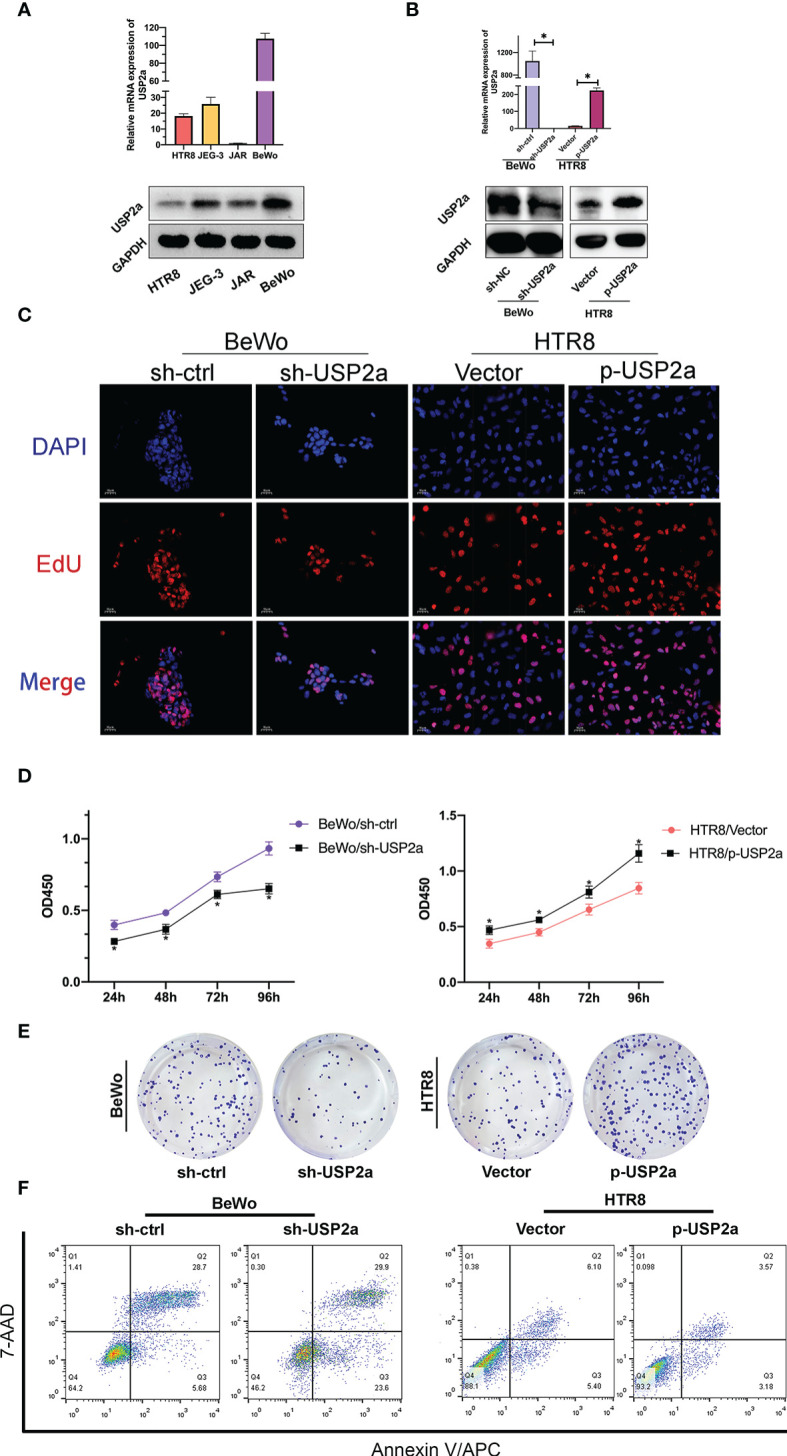
USP2a promoted human trophoblast‐derived cell lines proliferation and inhibited cell apoptosis *in vitro*. **(A)** Relative USP2a mRNA and protein expression levels in four human trophoblast‐derived cell lines were measured by RT‐PCR and western blot, respectively. GAPDH was used as the loading control. **(B)** Knockdown of USP2a by shRNA (sh‐USP2a) and upregulation of USP2a by plasmid expression were conducted in the BeWo and HTR8 cell lines. The mRNA expression and protein level of USP2a were confirmed by RT‐PCR and western blot. **(C)** Representative images of EdU labeling assay in p-USP2a/HTR8 and sh-USP2a/BeWo. DAPI staining blue; EdU staining red. Scale bar = 10 μm **(D)** Cell proliferation was measured 48 hours after transfection using the CCK8 assay. **(E)** Knocking down USP2a inhibits cell clone formation significantly in BeWo cells. Overexpression of USP2a promotes cell clone formation in HTR8 cells. **(F)** Quantitative flow cytometry measurements of apoptosis in trophoblast cell lines after transfection of either p-USP2a or shRNA. The percentage of live (7AAD−/Annexin V−), early apoptotic (7AAD−/Annexin V+), and dead (7AAD+/Annexin V+) cells was determined. *P < 0.05.

**Figure 3:**
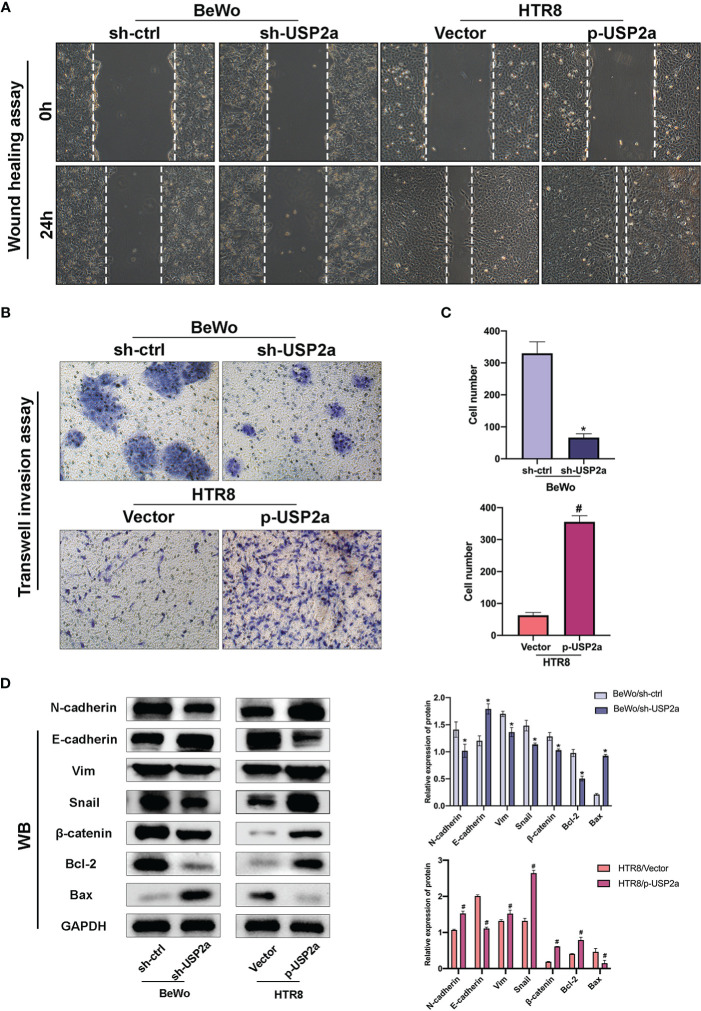
Effects of USP2a on the invasion and migration of trophoblast cells using the Transwell invasion assay and the wound-healing assay. **(A)** Wound healing scratches were imaged immediately and 24 h after initial scratch time to quantify relative migration. **(B)** After transfection, BeWo or HTR8 cells (in serum‐free medium) were seeded into inserts and incubated for 48 h for invasion assay. Cells at the lower surface of the membrane were counted and analyzed under a light microscope in five random fields. The staining intensity was quantified using the ImageJ software. Graphs show the means ± SD. **(C)** Western blot Graphs show the mean ± SD. Quantitative analysis of the average invasive cell numbers in each group. **(D)** The levels of E-cadherin, N-cadherin, Vimentin, Snail, β-catenin, Bax, and Bcl2 were measured using western blotting and quantitative analysis of the proteins was performed. *P < 0.05 *vs.* sh-Ctrl ^#^ *P* < 0.05 *vs* control vector, original magnification: ×100 **(A)** ×200 **(B)**.

To gain a deeper insight into the molecular function of USP2a, we first detected the mRNA expression of USP2a in the trophoblast cell lines, including HTR-8/SVneo, BeWo, JEG-3, and JAR. As shown in **Figure 2A**, we found that the relative level of USP2a in BeWo and HTR-8 cell lines was relatively higher and lower than those in the other two cell lines. Thereby, we chose these two cell lines for subsequent exploration. sh‐USP2a significantly decreased the expression of USP2a in BeWo, while p‐USP2a had the opposite effect in HTR8 **(** **Figure 2B** **)**. Following this, EdU labeling, CCK-8 analysis, and colony formation were performed to investigate the role of USP2a in trophoblasts proliferation. EdU assay revealed that knockdown of USP2a reduces the percentage of EdU-positive cells. Meanwhile, EdU positive cells increased due to USP2a overexpression **(** **Figure 2C** **)**. Comparable results of that higher USP2a level could induce trophoblasts proliferation were obtained from the CCK-8 assay **(** **Figure 2D** **)** and the colony formation assay **(** **Figure 2E** **)**. Apoptosis experiments indicated that knockdown of USP2a promotes apoptosis **(** **Figure 2F** **)**. We also observed decreased level of anti-apoptotic protein BCL2 in trophoblast cells when USP2a was knockdown, whereas the abundance of the pro-apoptotic protein BAX increased obviously. Opposite results were obtained in the USP2a overexpression cell line **(** **Figure 3D** **)**.

### USP2a Regulates the Invasion and Migration Ability of Trophoblast Cells

Next, the invasion and migration capacities of BeWo and HTR8 were estimated. Knockdown of USP2a inhibits trophoblast cell migration in imaging assay after the scratch test. In contrast, overexpression of USP2a exhibited a faster closure of the wound when compared to the control group **(** **Figure 3A** **).** When USP2a was knocked down, the numbers of invading cells were significantly decreased, whereas the numbers of invading cells were considerably raised when USP2a was overexpressed (**Figures 3B, C** **).** The results showed that knockdown of USP2a significantly inhibited trophoblasts migration and invasion compared with the control group, whereas overexpression of USP2a had an opposite effect. Considering the previous evidence, it seems that trophoblasts with increased migration and invasion capacity usually experience EMT (26). We analyzed several EMT markers by western blot **(** **Figure 3D** **)**. As expected, the overexpression of USP2a in trophoblasts induced an increase in the EMT phenotype. The knockdown of USP2a significantly inhibited EMT, as shown by the decreased expression of mesenchymal marker N-cadherin, Snail, and Vimentin and increased expression of epithelial marker E-cadherin, where overexpression of USP2a resulted in the opposite effects. Besides, we also observed an enhanced expression of β-catenin in USP2a-overexpression trophoblasts. Altogether, these results demonstrated that USP2a could regulate the invasion and migration ability of trophoblasts.

### Reduced USP2a Expression Impairs Nucleocytoplasmic Translocation of β-Catenin and Decreases Trophoblasts Invasion

**Figure 4:**
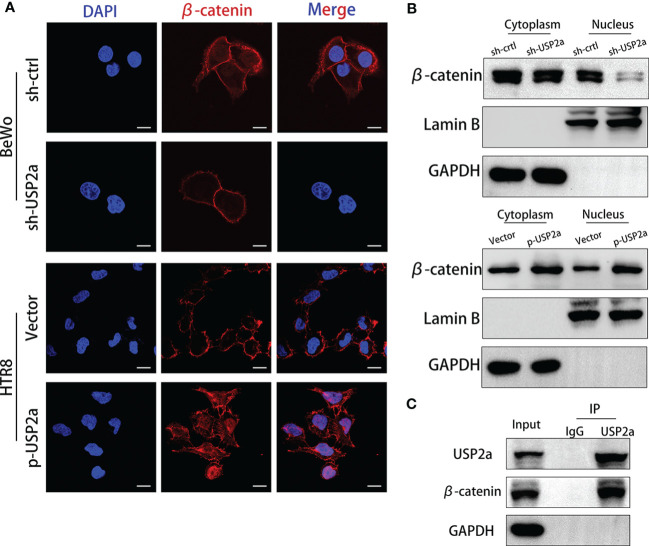
Reduced USP2a Expression impairs nucleocytoplasmic translocation of β-catenin. Knockdown of USP2a attenuates nuclear translocation of β-catenin in BeWo cells. Overexpression of USP2a induces nuclear translocation of β-catenin in HTR8 cells BeWo cells were transfected with sh-Ctrl or sh-USP2a, and HTR8 cells were transfected with p-USP2a or vector. Both cells were cultured for 48 hours. **(A)** The cells were seeded to immunofluorescent staining for β-catenin (red) and counterstained with DAPI (blue) to assess the subcellular localization of β-catenin and **(B)** western blot was performed to compare the levels of β-catenin in the nucleus and cytoplasm. GAPDH and lamin B were used as the loading control. **(C)** Co-IP assays of the interaction between USP2a and β-catenin in HTR8. Scale bar = 10μm.

**Figure 5:**
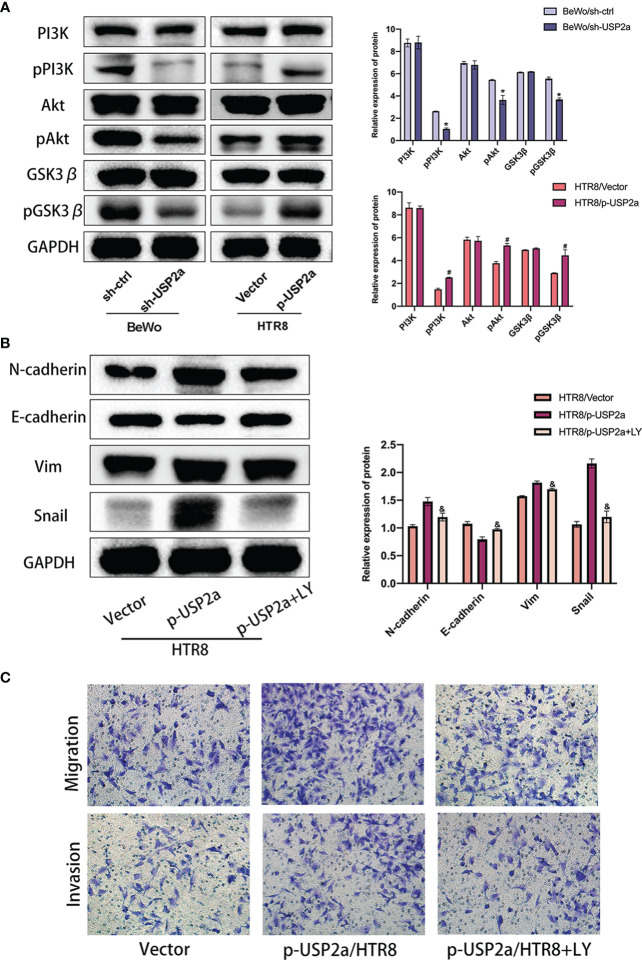
USP2a promotes EMT, migration and invasion of trophoblasts *via* activating PI3K/Akt/GSK3β pathway. **(A)** Western blot analyzes relative protein level of sh-Ctrl/BeWo, sh-USP2a/BeWo, Vector/HTR8, p-USP2a/HTR8. **(B)** Western blot analysis of sh-Ctrl/BeWo, sh-USP2a/BeWo, Vector/HTR-8, p-USP2a/HTR8 in the presence or absence of LY29004 (20 μM). **(C)** Migration and invasion capacity in sh-Ctrl/BeWo, sh-USP2a/BeWo, Vector/HTR8, p-USP2a/HTR8 in the presence or absence of LY294002 (20 μM) was determined by transwell system. Representative photographs of migratory and invasive cells (magnification, ×200) are shown. *P < 0.05, *vs.* sh-Ctrl ^#^ *P* < 0.05 *vs* vector. ^&^ *P* < 0.05 *vs* p-USP2a/HTR8.

β-catenin combines with the intracellular segment of E-cadherin to form a cadherin-catenin complex so that E-cadherin is located at the junction between cells maintaining cell adhesion (27, 28). If β-catenin accumulates in the cytoplasm, it will enter the nucleus from the cytoplasm and undergo nuclear translocation if the concentration reaches a certain level. In that case, β-catenin into the nucleus can activate the expression of downstream target genes, thereby promoting EMT (27). We used IF to detect the location and expression of β-catenin in HTR8 cells overexpressing USP2a and BeWo cells with USP2a knockdown. The results showed that the overexpression of USP2a significantly increased the expression level of β-catenin, especially the nuclear localization of β-catenin, while the knockdown of USP2a showed the opposite result **(** **Figure 4A** **)**. Then we further detected the expression of β-catenin in the cytoplasm and nucleus by Western blot, and the results were consistent with the IF results **(** **Figure 4B** **)**. we also detected the cooperation of USP2a and β-catenin in trophoblast cells by co-IP **(** **Figure 4C** **)**. Since the degradation of β-catenin is regulated by glycogen synthase kinase-3β (GSK-3β), and GSK-3β is a downstream molecule of the PI3K/Akt signaling pathway (29). Therefore, we suspect that USP2a may regulate EMT through the PI3K/Akt/GSK-3β signaling pathway. We detected the expression of the pathway proteins mentioned above when USP2a is overexpressed or downregulated **(** **Figure 5A** **)**. This confirmed our suspicions. p-USP2a/HTR8cells were treated with PI3K inhibitor LY294002, and then the changes of EMT-related indicators were detected by Western blot. We found that after LY294002 treatment, E-cadherin expression increased considerably, while the expression of N-cadherin, Vimentin, and Snail decreased significantly **(** **Figure 5B** **)**. Likewise, the invasion and migration ability were reduced to a degree in the p-USP2a/HTR8 cells **(** **Figure 5C** **)**.

### M2 Macrophage-Secreted TGF-β Cooperates With USP2a in Trophoblasts to Promote Invasion

**Figure 6:**
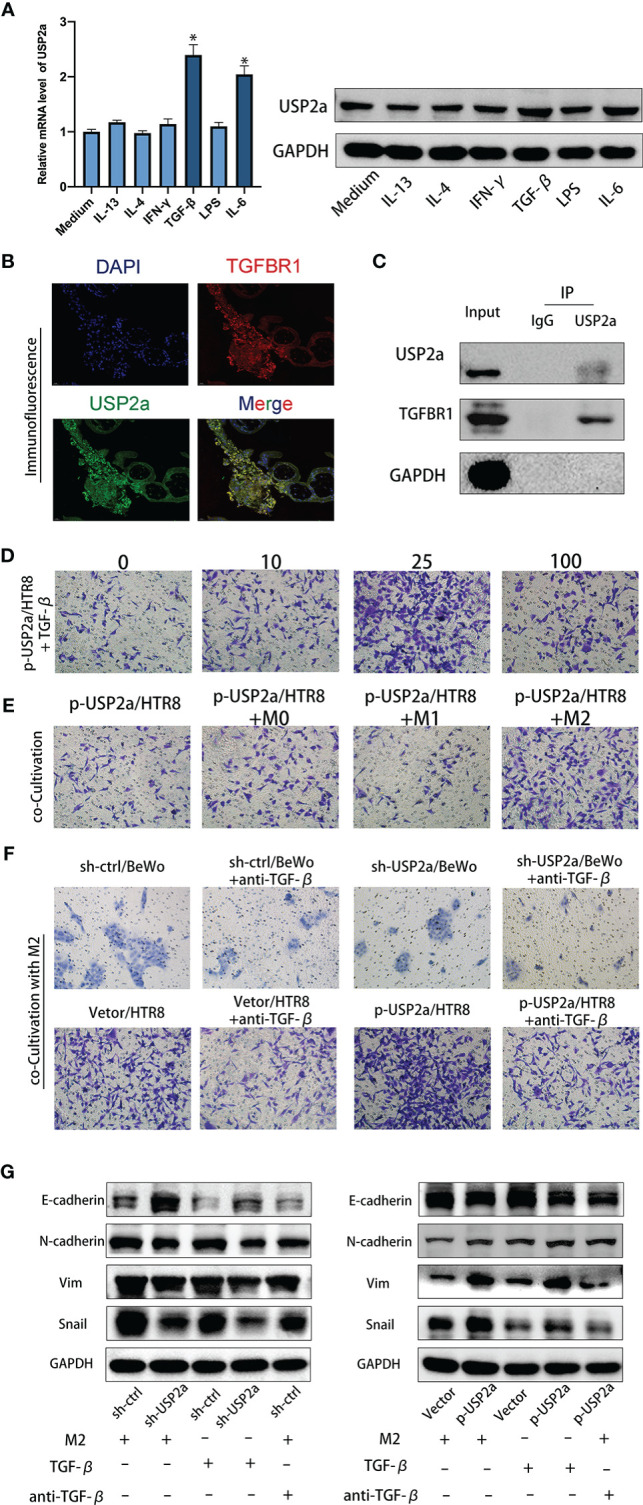
M2 Macrophage-secreted TGF-β cooperate with USP2a in trophoblasts to promote invasion. **(A)** Western blot and quantitative RT-PCR analysis of USP2a expression in HTR8 treated with IFN-γ, IL-13, IL-4, IL-6, LPS, or TGF-β for 24 hours. **(B)** Immunostaining for TGFBR1 and USP2a in villous from normal pregnancy woman (Scale bar = 20 μm). **(C)** co-IP assays of the interaction between USP2a and TGFBR1 in p-USP2a/HTR8. **(D)** Cell invasion capacity in HTR8 alone, HTR8 with 10ng/ml, 25ng/ml, or 100ng/ml rhTGF-β by transwell system. **(E)** Cell invasion capacity in p-USP2a/HTR8 alone, p-USP2a/HTR8 co-cultured with M0 macrophage, co-cultured with M1 macrophage, and co-cultured with M2 macrophage by transwell system. **(F)** Cell invasion capacity in sh-Ctrl/BeWo, sh-USP2a/BeWo, Vector/HTR8, p-USP2a/HTR8 co-cultured with M2 macrophage or above cells co-cultured with TGF-β depleted M2 macrophage were determined by transwell system, respectively. **(G)** Western blot assay of EMT proteins in sh-Ctrl/BeWo, sh-USP2a/BeWo, Vector/HTR8, p-USP2a/HTR8 co-cultured with M2 macrophage or above cells co-cultured with TGF-β depleted M2 macrophage were determined by transwell system, respectively. *P < 0.05, *vs.* HTR8. DAPI staining blue; TGFBR1 staining red; USP2a staining green. Representative photographs of invasive cells (magnification, ×200) are shown.

Previous studies reported that abnormal levels of various cytokines, including IL-4, IL-6, and IFN-γ regulated trophoblast growth and invasion in patients with RM (30). We wondered whether these factors also are involved in the regulation of USP2a expression in trophoblasts and work in concert to play a role in the pathology of RM. We stimulated HTR8 cells with different cytokines (IFN-γ, IL-13, IL-4, IL-6, LPS, or TGF-β) and then detected the relative expression levels of USP2a mRNA and protein by RT-PCR and Western blot. The results showed that the expression of USP2a increased significantly after IL-6 and TGF-β stimulation, but there was no significant change after IFN-γ, IL-13, IL-4, and LPS stimulation **(** **Figure 6A** **)**. Since our group and others have confirmed the effect of IL-6 on trophoblasts in the early stage of pregnancy (16, 30), we will mainly explore the effect of TGF-β. We also detected TGF-β expression in trophoblast cell lines that were transfected with a USP2a-overexpressing plasmid or control vector. No significant change was observed in TGF-β expression in USP2a-expressing cells in RT-PCR analysis. Next, we detected the TGF-β receptor (TGFBR1) on trophoblasts in the placental villous tissues and found the colocalization of TGFBR1 and USP2a by co-immunofluorescence staining **(** **Figure 6B** **)**. Therefore, we postulated that USP2a affects trophoblasts invasion by interacting with TGFBR1. To test this assumption, we conducted co-IP experiments. Co-IP experiments further corroborated the interaction between USP2a and TGFBR1 **(** **Figure 6C** **)**. We check the invasion of p-USP2a/HTR8 treated with exogenous rhTGF-β. At a concentration of 25ng/ml of TGF-β, p-USP2a/HTR8 exhibited the most invasion ability **(** **Figure 6D** **)**. TGF-β is one of the important cytokines produced by M2 phenotype macrophages in the fetal-maternal interface (13). p-USP2a/HTR8 were co-cultivated with M0, M1, and M2 macrophages separately. Compared with co-cultivation with M0 macrophages, co-cultivation with M2 macrophages significantly increased trophoblast invasion **(** **Figure 6E** **)**. This effect is more evident in the USP2a-upregulating HTR8 cells. Intriguingly, when neutralizing antibody for TGF-β was added, the invasion ability of trophoblasts promoted by M2 was alleviated. A similar effect was confirmed in USP2a-downregulated BeWo cells **(** **Figure 6F** **).** The relative expression evaluated by western blotting showed similar effects **(** **Figure 6G** **).** These results strongly suggested that M2 macrophage-secreted TGF-β may work together with USP2a as one of the regulators of the biological functions of trophoblasts in patients with RM.

## Discussion

It is generally recognized that placental insufficiency is one of the main culprits in RM arising from shallow trophoblast invasion at the initial stages of pregnancy (31, 32). The present study illustrated that USP2a is low‐expressed in the placental villous samples of RM women, and aberrant expression of USP2a can impact trophoblast invasion by regulating the EMT process. Further research showed that USP2a regulates β-catenin translocating into the nucleus through PI3K/AKT/GSK3β pathway, thereby inducing trophoblasts migration and invasion. Moreover, TGF-β secreted by M2 macrophages could interact with USP2a in trophoblasts to regulate placentation.

Ubiquitination describes the process during which the 76-residue protein termed ubiquitin is covalently conjugated to intercellular proteins (17). Ubiquitination is reversible and precisely regulated due to the activity of deubiquitinating enzymes (18). Together these two systems collaborate as control of cell signaling networks by regulation of protein interactions and activities. USP2a, a cysteine protease, as a member of the ubiquitination specific protease family, is extensively expressed in many human cancers and often correlates with tumor progression and poor prognosis. Previous studies suggest USP2a interacts with FAS which often overexpresses in biologically aggressive human tumors.

Further, functional inactivation of USP2a results in decreased FAS protein with reinforced apoptosis of prostate cancer cells (33). Also, Priolo et al. report that USP2a overexpression in prostate cancer contributes to tumorigenesis by repressing p53 (34). Allende-Vega further illustrated this mechanism that ectopic expression of USP2a causes accumulation of Mdm2 and promotes Mdm2-mediated p53 degradation (35). USP2a could promote hepatocellular carcinoma invasion *via* deubiquitinating RAB1A (36). These studies indicated USP2a presented oncogenic properties (34, 37, 38). Since the processes of trophoblast proliferation, migration, invasion, and apoptosis at the implantation site share similar biochemical mediators of malignant tumor metastasis (39). Therefore, we examined the role of USP2a in placentation during early pregnancy. The results of our study indicate USP2a could promote trophoblast invasion, migration, and proliferation as well as reduce apoptosis. Thus, we illustrate the critical role of USP2a in the pathogenesis of RM by affecting the invasion and migration of trophoblasts.

We also observed that overexpression of USP2a resulted in upregulation of β-catenin and downregulation in E-cadherin. E-cadherin/β-catenin complex located in cell-cell adherent junctions in the cell membrane creates tight cell-cell interactions, thus restraining cell mobility and maintaining epithelial integrity (27, 28). However, decreased E-cadherin expression results in degradation of the E‐cadherin/β‐catenin conjunction. Therefore, β-catenin is released into the cytoplasm of trophoblasts, and next translocated from the cytosol into the nucleus and induces EMT genes transcription (40–42). Accordingly, we evaluated the level of β-catenin nuclear translocation. The overexpression of USP2a promoted the nuclear transfer of β-catenin in trophoblast cells. Instead, the knockdown of USP2a suppressed this process. Unsurprisingly the trophoblasts acquired mesenchymal-cell markers prone to migration and invasion, such as N-cadherin, vimentin, and Snail. This is consistent with previous literature that the moderate β-catenin signaling pathway promotes the invasion of EVTs and provides the basis for successful embryo implantation (43). Our results also suggest that USP2a interreact with β-catenin. As USP2a has been reported to deubiquitinate and stabilize to promote β-catenin nuclear accumulation and transcriptional activity of β-catenin (24). We believe USP2a could enhance trophoblast invasion by both PI3K/Akt/GSK3β/β-catenin pathways and deubiquitinating β-catenin.

**Figure 7:**
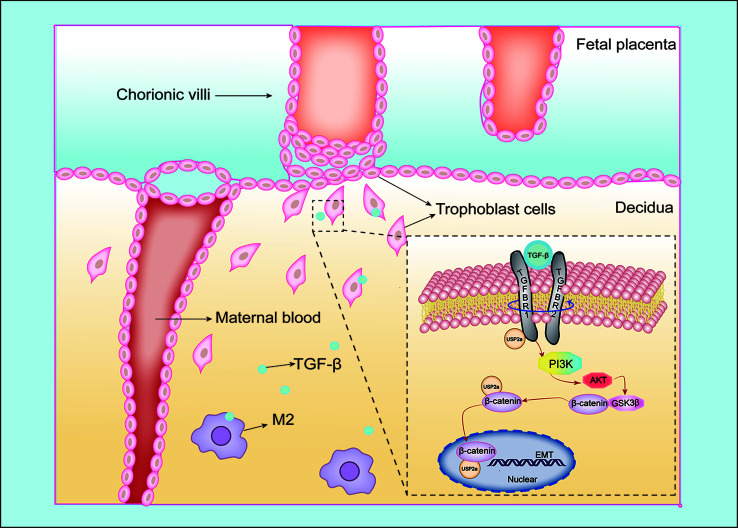
Schematic diagram of the interaction between trophoblasts and macrophages at the maternal-fetal interface. Our study illustrated USP2a facilitates the EMT programme to enhance migration and invasion of trophoblasts secreted by phosphorylating PI3K/Akt/GSK3β/β-catenin signaling pathways. In the meantime, USP2a could interreact with β-catenin as a “shortcut” to promote nuclear β-catenin accumulation. TGF-β secreted by M2 macrophage could work together with USP2a to promote trophoblast invasion. Thereby, USP2a participates in regulating the establishment and maintenance of normal pregnancy.

The regulation of the immune system during pregnancy is a fascinating and complex mechanism. Interactions between the maternal stromal components and fetal-derived trophoblasts of the uterus and placenta are not an isolated occurrence but rather are under a superimposition layer of regulation by the maternal immune cells that populate the decidua (12). On the one hand, embryo implantation requires a local inflammatory environment that promotes cell clearance, angiogenesis, and cell growth (9). On the other hand, the fetus as a semi-allograft that carries paternal antigen would be rejected if there were no tolerance environment of the maternal immune system during pregnancy (44). Macrophages represent the second most leukocyte subsets in the human maternal-fetal interface. The polarization of decidual macrophages is biased to the M1 phenotype during the pre-implantation period. Then they begin to transition to a mixed M1/M2 profile when the trophoblasts establish attachment into the endometrium and invade the uterine stroma. In general, decidual macrophages are characterized by an immunosuppressive phenotype as M2 polarization secreting IL-10, TGF-β, and G-CSF, supporting fetal-maternal immune tolerance during implantation (45, 46). Abnormal macrophage polarization is related to many pregnancy complications, such as PE, premature birth, and RM (47). The previous study has shown that TGF-β can increase the invasiveness of placental cells in rats (48). In our study, TGF-β promoted trophoblast invasion in a dose-dependent manner. At a concentration of 25ng/ml of TGF-β, trophoblast cells exhibited the most invasion ability, while 100ng/ml of TGF-β shows little pro-invasive function in trophoblast. A previous study revealed the expression of TGF-β in pregnant rats is increased following implantation (days 5.5 to 6.5) and is maximal during regression of the decidual basil (day 14) (49). These results corroborate the findings of previous work that shows TGF-β can also restrict trophoblasts invasion in late pregnancy to prevent preeclampsia (50). We assume that the regulation of TGF-β on trophoblast is under complicated mechanisms through the whole pregnancy period. Here we illustrated USP2a collaborates with TGF-β to promote trophoblast cell invasion to help maintain normal pregnancy. Our results also demonstrate USP2a could interreact with TGFBR1. Therefore, we speculated that during normal pregnancy, decidua macrophages present an M2 phenotype and secrete TGF-β as crosstalk between decidua macrophages and the trophoblast cells. Meanwhile, TGF-β could promote the USP2a expression of trophoblast cells. USP2a could further interreact with TGFBR1, thus improving trophoblast invasion ability **(** **Figure 7** **)**. However, the exact mechanism remains unclear and needs further research.

Chaiworapongsa reported a case that a 38-year-old RM patient who took the histopathological examination of the placenta from a previous pregnancy showed fibrinoid deposition in the intervillous space surrounding more than 50% of the villi in some full-thickness sections. After receiving statin therapy, the patient delivered a neonate without major complications vaginally at 34 weeks of gestation (51). An array-based kinase assay spanning the entire human kinome indicated statins induce upregulation of PTEN activity leading to downregulation of the PI3K/Akt signaling (52). The PI3K/Akt signaling pathway is one of the major driving forces in a range of cellular functions and dysregulation of PI3K/Akt has been implicated in many human diseases including RM (53). Interestingly, we also detected USP2a expression in the glandular epithelium and stromal cells of human endometrial tissue and decidua tissue. When human endometrial stromal cells were cultured and stimulated by progesterone and cAMP, levels of USP2a were increased. In comparison, decidualization was suppressed markedly silencing USP2a. This suggests that progesterone and cAMP may modulate USP2a production and that USP2a may contribute to regulating decidualization. Together we can assume that USP2a plays an essential role in early pregnancy.

Several limitations need to be pointed out regarding the current study. First, whether USP2a has an impact on regulating macrophage polarization still requires further research. Second, in this study, we exclusively focused attention on the effects of macrophages but ignored other immune cells. Numerous immune cells, cytokines, chemokines, and signal molecules from the maternal-fetal interface microenvironment work synergistically to establish and maintain pregnancy. Finally, the findings from this study came from the clinical tissue and *in vitro* verification, which needs further identification on *in vivo* models. These limitations refer that these findings of the present study need to be interpreted cautiously.

In summary, this study provides new insights into the distinctive trophoblast proliferation and invasion events during the early stage of human pregnancy. Furthermore, the evidence from this study suggests transplacental regulation of cellular immunity and a possible regulating mechanism of TGF-β secreted by macrophages in regulating trophoblast invasion. These novel findings have important implications for the role of trophoblast invasion in maintaining a successful pregnancy.

## Data Availability Statement

The original contributions presented in the study are included in the article/supplementary material. Further inquiries can be directed to the corresponding authors.

## Ethics Statement

The studies involving human participants were reviewed and approved by The Ethics Committee of Renmin Hospital of Wuhan University. The patients/participants provided their written informed consent to participate in this study.

## Author Contributions

Conceptualization, JW and JD. Data curation, JW. Formal analysis, SZ. Investigation, JW, XC, and SZ. Methodology, JD. Project administration, JW and SY. Supervision, JD. Validation, XC and JD. Visualization, JD and SY. Writing – original draft, JW. Writing – review and editing, TY and YZ. All authors contributed to the article and approved the submitted version.

## Funding

This work was supported by the following grants: National Key Research and Development Program of China (No. 2018YFC1004601), the National Natural Science Foundation of China (No. 81801540, 81771662) and the Fundamental Research Funds for the Central Universities (2042021kf0082)

## Conflict of Interest

The authors declare that the research was conducted in the absence of any commercial or financial relationships that could be construed as a potential conflict of interest.

## Publisher’s Note

All claims expressed in this article are solely those of the authors and do not necessarily represent those of their affiliated organizations, or those of the publisher, the editors and the reviewers. Any product that may be evaluated in this article, or claim that may be made by its manufacturer, is not guaranteed or endorsed by the publisher.
